# The Cytotoxic Effects of Cannabidiol and Cannabigerol on Glioblastoma Stem Cells May Mostly Involve GPR55 and TRPV1 Signalling

**DOI:** 10.3390/cancers14235918

**Published:** 2022-11-30

**Authors:** Tamara T. Lah, Bernarda Majc, Metka Novak, Ajda Sušnik, Barbara Breznik, Andrej Porčnik, Roman Bošnjak, Aleksander Sadikov, Marta Malavolta, Selma Halilčević, Jernej Mlakar, Roby Zomer

**Affiliations:** 1 Department of Genetic Toxicology and Cancer Biology, National Institute of Biology, 1000 Ljubljana, Slovenia; 2 Jožef Stefan International Postgraduate School, Nanosciences and Nanotechnologies, 1000 Ljubljana, Slovenia; 3 Department of Neurosurgery, University Medical Centre Ljubljana, 1000 Ljubljana, Slovenia; 4 Faculty of Computer and Information Science, University of Ljubljana, 1000 Ljubljana, Slovenia; 5 Institute of Pathology, Faculty of Medicine, University of Ljubljana, 1000 Ljubljana, Slovenia; 6 MGC Pharmaceuticals d.o.o., 1000 Ljubljana, Slovenia

**Keywords:** glioblastoma, glioma, cannabigerol, cannabidiol, cannabinoid receptors, stem cells

## Abstract

**Simple Summary:**

In glioblastoma (GBM), the highest-grade IV glioma, members of the large membrane receptor family of G protein-coupled receptors (GPCRs) are upregulated including CB1 and GPR55 that along with non-selective ion transporter protein TRPV1 bind endocannabinoids and phytocannabinoids with different affinities and signalling effects. The receptors are part of the endocannabinoid system (ECS) and are overexpressed in glioma to raise cancer defence signalling by ESC in tumour cells. We demonstrated that *GPR55* and *TRPV1* genes, but not CB1/*CNR1* genes, correlated to GBM stem cell (GSC) gene markers, and both were highly expressed in GSCs compared with differentiated GBM cells. Therefore, we propose that GPR55 and TRPV1 receptors are the best targets for the antagonistic cannabinoids CBD and CBG (in an optimized mixture) to eliminate GBM stem cells. This approach avoids using psychoactive THC, which is potentially harmful, particularly in older GBM patients, and should be further tested in animal experiments and clinical trials.

**Abstract:**

Glioblastoma (GBM) is one of the most aggressive cancers, comprising 60–70% of all gliomas. The large G-protein-coupled receptor family includes cannabinoid receptors CB1, CB2, GPR55, and non-specific ion receptor protein transporters TRPs. First, we found up-regulated *CNR1, GPR55*, and *TRPV1* expression in glioma patient-derived tissue samples and cell lines compared with non-malignant brain samples. *CNR1* and *GPR55* did not correlate with glioma grade, whereas *TRPV1* negatively correlated with grade and positively correlated with longer overall survival. This suggests a tumour-suppressor role of *TRPV1*. With respect to markers of GBM stem cells, preferred targets of therapy, *TRPV1* and *GPR55*, but not *CNR1*, strongly correlated with different sets of stemness gene markers: *NOTCH, OLIG2, CD9, TRIM28*, and *TUFM* and *CD15*, *SOX2, OCT4*, and *ID1*, respectively. This is in line with the higher expression of *TRPV1* and *GPR55* genes in GSCs compared with differentiated GBM cells. Second, in a panel of patient-derived GSCs, we found that CBG and CBD exhibited the highest cytotoxicity at a molar ratio of 3:1. We suggest that this mixture should be tested in experimental animals and clinical studies, in which currently used Δ9-tetrahydrocannabinol (THC) is replaced with efficient and non-psychoactive CBG in adjuvant standard-of-care therapy.

## 1. Introduction

The impact of cancer in the world is evident from the increasing number of new cases in the younger population and the increasing prevalence of the disease; however, mortality is decreasing due to early detection and more efficient therapies, making cancer a chronic disease [1,2]. However, this is not the case with glioblastoma (GBM), the most malignant glioma of the highest stage. GBMs account for 60–70% of astrocytic brain cancers and have an incidence of 5–7 cases in 100.000 inhabitants in Western countries [3] and a median survival period of only 14–16 months [4,5]. This tumour is among the most aggressive and therapeutically non-responsive of all cancers. Based on molecular classification by The Cancer Genome Atlas (TCGA), we can distinguish three GBM subtypes: proneural (PN), classical (CL), and mesenchymal (MES). The markedly different transcriptional profiles of the three GBM subgroups affect clinical prognosis, with PN having a slight survival advantage vs. more aggressive MES [6]. However, gene expression and single-cell RNA analyses of different regions in the same tumour have shown that the molecular subtypes may coexist [7], reflecting intra-tumour heterogeneity, which is partially responsible for the high resistance of GBM to therapy.

The second reason for the permanent resistance of GBM to therapy is glioma-initiating cells, named GBM stem cells (GSCs) [8], which represent only a fraction of most resistant cancer cells. GSC numbers increase with glioma progression and are the most abundant in grade IV GBM. The GSC phenotype results from oncogenic mutations of transformed neural stem cell progenitors and/or the trans-differentiation of astrocytes and other brain cells [9] through the enhanced expression of genes that regulate GBM cell “stemness” [10]. The characteristic slow proliferation, enhanced DNA repair, and multidrug resistance mechanisms of GSCs enable their survival after radio- and chemotherapy and facilitate tumour regrowth, leading to relapses and mortality. [11]. As reviewed by Alves et al. [11], adjuvant treatments with natural products, e.g., cannabinoids, can decrease GSC viability and thus show promise for eliminating GSCs and preventing tumour recurrence. Based on current knowledge, cannabinoids and synthetic ligands of their receptors target GSCs and their stemness-related signalling pathways, forcing them to differentiate [12,13,14].

Phytocannabinoids have been studied more intensively over the past 30 years, and evidence has accumulated regarding their efficient cytotoxicity on cancer stem cells via signalling mechanisms (summarized by Dumitru et al. [15]) as targeting the “hallmarks of cancer” [16]. Cannabinoids comprise plant-derived terpenophenols, which are divided into 10 subclasses according to their structural features [17].

During phytocannabinoid biosynthesis, their precursor cannabigerol (CBG) is transformed to Δ9-tetrahydrocannabinol (THC) and cannabidiol (CBD), most abundant in the flowers of plants, followed by a number of minor cannabinoids. Thus, being present in much lower content (<10%) [18], CBG’s effects on pathophysiological processes, especially cancer, have only recently gained more attention and understanding of its binding to known cannabinoid receptors (CNRs) [19].

CNRs were discovered about two decades ago, yet their signalling is still not completely understood [20]. The phenotypic effects and underlying mechanisms of the signalling of single and combined administrations of cannabinoids, of which mostly THC and CBD have been studied, have recently been reviewed in extensive research [21]. There are two major families of cannabinoid receptors: G-protein-coupled cannabinoid receptors (GPCRs) and transient receptor potential channels (TRPs). GPCRs are known to play a crucial role in various cancers and are common therapeutic targets in GBM [22], e.g., tyrosine receptor kinases, epidermal growth factor receptors, and platelet-derived growth factor receptors. GPCRs are linked to heteromeric G-proteins, i.e., Gα, Gβ, Gγ, and their subtypes, leading to various highly specialized downstream signalling cascades. Among GPCRs, the two CNRs CB1 and CB2 are most abundant in the human central nervous system and immune system, respectively [23], and their levels are elevated in cancer cells. Genetically distinct CB3, also known as GPR55, is also overexpressed in many cancers, including GBM [24]. TRPs comprise non-selective cation channels that are ubiquitously expressed in mammalian tissues [25]. There are seven TRP subfamilies with different structure-dependent biophysical properties [25,26]. The TRP vanilloid (TRPV) family includes homo- or hetero-tetrameric calcium channels, such as TRPV1 [27,28] and TRPV2, which are imbedded in the plasma membrane and endoplasmic reticulum (ER) [26]. TRPV1 oncogenic activity [24] is linked to cancer-driven pain [29].

A large body of research accumulated on the differential binding of cannabinoids and endocannabinoids (eCB) to CB1 and/or CB2 in vitro, where THC was found to bind stronger to CB1 than to CB2. In contrast, CBD binds stronger to CB2. Similar to CBG, which binds with much lower affinity to both receptors. Yet they may not bind to identical orthostatic site as does THC; thus, CBD and CBG are actually CB1-negative allosteric modulator slightly reducing THC binding in combined application. CBD and CBG are also both GPR55 antagonists to its oncogenic signalling. CBG more than CBD binds and dilates transmembrane TRPV1 Ca^2+^ permeable pores and thus more or less desensitizes TRPV1 and perturbs its signalling activity [27] in high grade astrocytoma [30] and NSCs [31]. Yet CBG and CBG’s differential binding affinity to the three receptors affect their specific signalling in GBM and GSC cells [13,32,33].

We were the first to report the effects of CBG on GBM and to demonstrate that CBD and CBG, both alone and in combination, induce caspase-dependent cell apoptosis, with no additive THC effect [34]. CBG also inhibited GBM cell invasion in a similar manner to CBD. We have demonstrated that the combination of sub-cytotoxic concentrations of CBG and CBD reduced GBM cell viability in an additive manner that which was more efficient than with THC. The focus of the present research was to first determine CB1/CNR1, GPR55, and TRPV1 gene expression in glioma samples and in differentiated GBM cells and GSCs. We aimed to correlate receptor expression with GBM heterogeneity and particularly GBM stemness. By optimizing the CBG:CBD ratio, we further aimed to achieve better cytotoxic efficacy on GSCs in vitro, using a panel of patient-derived GBM and GSC lines that represent the desired target of precision therapy.

## 2. Materials and Methods

### 2.1. Cannabinoids

Purified CBD and CBG extracts from the cannabis plant were provided by MGC Pharmaceuticals [34]. For GBM cell toxicity tests, CBD and CBG were prepared in base emulsion that is the subject of a patent application.

### 2.2. Tissue Samples and Cell Lines

Glioma surgical biopsies were obtained from patients who underwent surgery at the Department of Neurosurgery, University Medical Centre Ljubljana, Slovenia. Altogether, 89 de novo GBM samples, 6 recurrent GBMs, and 17 grade I and II gliomas were obtained. As a reference, we also obtained 16 samples of non-cancerous brain tissues. The study was approved by the National Medical Ethics Committee of the Republic of Slovenia (approval No. 0120-179 190/2018/26). Patients’ biological material as well as primary cell cultures were prepared at National Institute of Biology as described previously [34] and are, in addition to corresponding relevant clinical and histopathological data (information), deposited in Slovenian Glioblastoma Bank (GLIOBANK).

The established differentiated human GBM cell line U373 was purchased from the American Type Culture Collection (Manassas, VA, USA) and maintained in high-glucose Dulbecco’s modified Eagle medium (GE Healthcare, Chicago, IL, USA), supplemented with 10% (*v*/*v*) heat-inactivated foetal bovine serum, 2 mM L-glutamine, 100 IU/mL penicillin, and 100 µg streptomycin. The GSC line NCH644 was purchased from Cell Lines Service (GmbH, Eppelheim, Germany). These and patient-derived GSCs were grown as spheroid suspensions in complete neurobasal medium, 20 ng/mL basic fibroblast growth factor, and epidermal growth factor (all from Invitrogen, Life Technologies, Carlsbad, CA, USA). All cell lines were maintained at 37 °C with 5% CO_2_ and 95% humidity and were tested for mycoplasma contamination using the MycoAlert Mycoplasma Detection Kit (Lonza Pharma & Biotech Ltd., Bend, OR, USA).

### 2.3. Gene Expression Analysis

qPCR analysis of gene expression and GBM subtype markers was performed as described by Lah et al. [34] and Novak et al. [35]. Briefly, total RNA from GBM tissues and cells was isolated using AllPrep DNA/RNA/Protein Mini Kit (Qiagen, Hilden, Germany) according to the manufacturer’s instructions. RT-qPCR was performed with FAM-MGB probes with Fluidigm BioMark HD System Real-Time PCR (Fluidigm, South San Francisco, CA, USA) using 48.48 Dynamic Arrays IFC [36]. Relative mRNA copy numbers were normalized to the housekeeping genes *HPRT1* and *GAPDH*. The assays are described in Table S1.

### 2.4. Cell Viability Assay

Cell viability was determined as described before [34] with the MTT assay, i.e., 3-(4,5-dimethylthiazol-2-yl)-2,5-diphenyltetrazolium-bromide (Sigma-Aldrich, St. Louis, MO, USA) for GBM differentiated cells and with MTS 3-(4,5-dimethylthiazol-2-yl)-5-(3-carboxymethoxyphenyl)-2-(4-sulfophenyl)-2H-tetrazolium salt (Promega, Madison, WI, USA) for GSCs. Differentiated GBM cells and GSCs were seeded onto 96-well plates at densities of 5000 and 8000–10000 cells/well, respectively. Cells were treated with different concentrations (0.32–320 µM) of the cannabinoids CBG and CBD alone or combined at the molar ratio CBD:CBG 3:1 (7.6:2.5–245:80 µM) with 100 µM temozolomide (Sigma-Aldrich, St. Louis, MO, USA). Cell viability was measured after 48 h of incubation by adding MTT or MTS reagent. All dose–response experiments were performed in triplicate, i.e., with three biological repeats. Absorbance was measured as the change in optical density (ΔOD 570/690 nm) using a microplate reader (Synergy™ HT, Bio-Tec Instruments Inc., Santa Clara, CA, USA). Cell viability and IC50 µM values were calculated using dose–response curves in GraphPad Prism software. Controls assays contained only the base emulsion (0.1%) and DMSO (≤ 0.4%, *v*/*v*) without CBD and CBG. Cell viability was measured after 48 h of incubation by adding MTT or MTS reagent. All dose–response experiments were performed in triplicate, i.e., with three biological repeats, vs. h. Absorbance was measured as the change in optical density (ΔOD 570/690 nm) using a microplate reader (Synergy™ HT, Bio-Tec Instruments Inc., Santa Clara, CA, USA). Cell viability and IC50 µM values were calculated using dose–response curves in GraphPad Prism software.

### 2.5. Statistical Methods

To determine the associations between *GPR55*, *CNR1*, and *TRPV1* mRNA expression and clinical variables, i.e., patient survival, death, and tumour grade, Pearson correlation coefficient analysis was performed. The correlations between patient characteristics and *GPR55*, *CNR1* and *TRPV1* mRNA levels were evaluated. Overall survival (OS) was defined as the period of time (in months) from the date of diagnosis to the date of death (event) or last follow-up (censored data). In the absence of any meaningful or predefined cut-offs, first (Q1), second (Q2; median), and third (Q3) quartiles were used as cut-off values for low and high gene expression levels for this cohort of patients. OS was estimated by the Kaplan–Meier methodology, and the log-rank test was used to compare different categories. A *p*-value below 0.05 was considered statistically significant. All reported *p*-values are two-tailed. All statistical analyses were performed using SPSS (version 21, Chicago, IL, USA).

## 3. Results

### 3.1. CNR1 mRNA Levels Are Increased in GBM Tissue Samples

**Figure 1:**
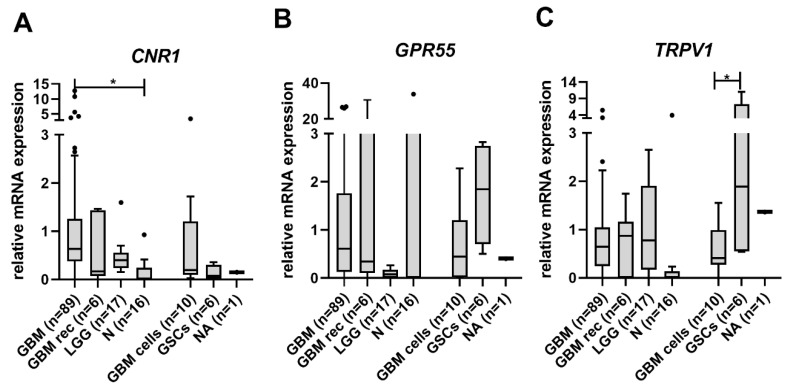
Receptor mRNA levels in different types and grades of glioma samples. The expression of (**A**) *CNR1* (CB1), (**B**) *GPR55*, and (**C**) *TRPV1* in glioma, non-cancerous brain tissues, and GBM cells. mRNA values were normalized to the housekeeping genes *HPRT1* and *GAPDH* and analysed with quantGenius software (developed at NIB). *n*: number of samples; *N*: non-cancerous brain tissues; LGG: grade I or II gliomas (pilocytic astrocytoma, astrocytoma, oligodendroglioma); GBM: glioblastoma; GBM rec: recurrent glioblastoma; GBM cells: primary glioblastoma cells; GSCs: glioblastoma stem cells isolated from patient tumour samples; NA: normal astrocytes versus N: the non-cancerous brain tissues (* *p* < 0.05).

We analysed *CNR1*, *GPR55*, and *TRPV1* mRNA levels in tissue samples from normal non-malignant brain tissues (*n* = 16) and neoplastic brain tissues: low-grade (WHO grades I and II) glioma (*n* = 17), WHO grade IV glioma (wild-type GBM) (*n* = 89), and recurrent GBM (*n* = 6), as well as from primary GBM cells (*n* = 10) and GSCs (*n* = 6) isolated from patient tumour samples. *CNR1* levels were significantly higher in GBM samples compared with non-cancerous brain tissues (Figure 1A). *GPR55* expression was higher in recurrent GBM; however, the sample number was small (*n* = 6) and with large diversity (Figure 1B). *TRPV1* levels were significantly higher in GSCs compared with GBMs (Figure 1C). Most interesting are the comparisons of receptor expressions in GBM vs. GSC cells. Despite low sample numbers, GSCs exhibit lower *CNR1* expression, higher *GPR55* expression, and significantly higher *TRPV1* expression.

### 3.2. Lower TRPV1 mRNA Levels Indicate Shorter Glioma Patients’ Survival

**Figure 2:**
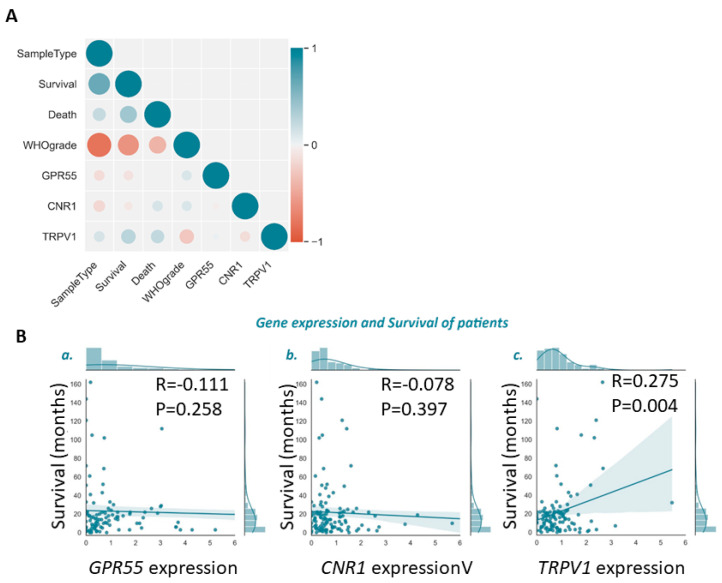
Correlation of cannabinoid receptors with glioma patient survival. (**A**) Graphical representation of the correlation matrix for the correlations between the expression of the receptors *GPR55* (*n* = 113), *CNR1* (*n* = 129), and *TRPV1* (*n*= 116) and glioma sample type and overall glioma patient survival (months). The correlations range from −1 (red dots) to 1 (blue dots). The closer the values are to 0, the more uncorrelated the variables are. (**B**) Estimation of the regression model between patient survival and the expression of three cannabinoid receptors: *GPR55* (**a**.; *n* = 106.), *CNR1* (**b**.; *n* = 120), and *TRPV1* (**c**.; *n* = 109). R: the correlation coefficient between the analysed variables; *p*: statistical *p*-value (considered significant when *p* < 0.05).

We analysed the correlations between cannabinoid receptor mRNA expression and two clinical variables: glioma WHO grades II–IV and overall survival in 120 glioma samples (Figure 2). Significant correlations were only found between survival and TRPV1 expression (Figure 2B): that lower TRPV1 mRNA expression correlates with shorter glioma patient survival.

The results in Figure 2 show that *CNR1* and *GPR55* expression correlate poorly and non-significantly with glioma malignancy and patient survival. Conversely, the *TRPV1* gene was significantly downregulated with glioma progression to GBM (Figure 2, Table 1). However, its expression did not significantly differ between low-grade glioma, GBM, and recurrent GBM (Figure 1C).

### 3.3. Cannabinoid Receptors Expression and Association with Survival of GBM Patient Survival

**Figure 3:**
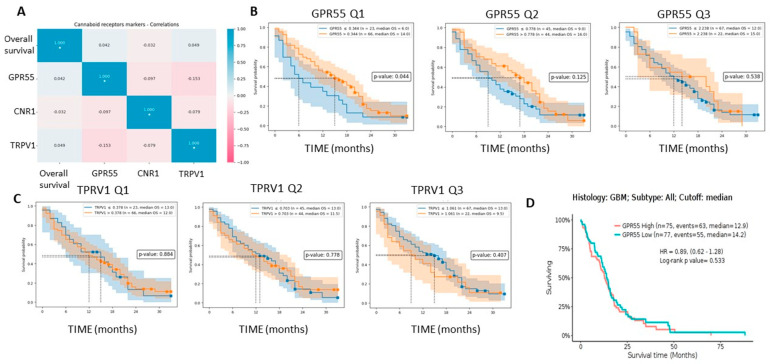
Correlation matrix and overall survival curves for cannabinoid receptor expressions in GBMs. (**A**) Correlation matrix data show positive significance between GBM patient (*n* = 89) survival and *GPR55* (*p* = 0.042) and *TRPV1* (*p* = 0.049) receptor gene expressions. (**B**,**C**) Kaplan–Meier survival curves of GBM patients (*n* = 89) stratified by three cut-off levels of *GPR55* (**B**) and *TRPV1* (**C**) mRNA expression. (**D**) Kaplan–Meier survival curves of GBM patients, as analysed from the TCGA data bank GLIOVIS with GBM patients (*n* = 152) with *GPR55* mRNA expression level data. Survival at median value was not significant. GBM: glioblastoma. * *p* < 0.05.

The expressions of cannabinoid receptors CNR1, GPR55, and TRPV1 were also compared in 89 samples of WHO grade IV GBM (IDH wild type) (Figure 3). In our cohort of 89 GBM tissues, there were no correlations among the three receptors mRNA levels. There was also no (for CNR1 and TRPV1) or poor (for GPR55) correlation between cannabinoid receptor expression and overall patient survival (Figure 3A). Kaplan–Meier analyses of TRPV1 and GPR55 at three cut-off values (the two quartiles and median) showed that patients with higher first quartile GPR55 levels have significantly better survival rates (*p* = 0.044) (Figure 3B), whereas no significance was observed for TPRV1 (Figure 3C). GPR55 at the median cut-off was also validated by an independent cohort of grade IV GBM patients from the GLIOVIS data bank and was also determined to be non-significant (Figure 3D).

Of note, all GBM tissues were collected during the first surgery of still untreated patients; however, the survival rates relate to treatment. Thus, the survival curves actually mirror the response to standard-of-care protocols.

### 3.4. Cannabinoid Receptors Expression Correlate with GSC Biomarkers Expression in GBM Tissues

**Figure 4:**
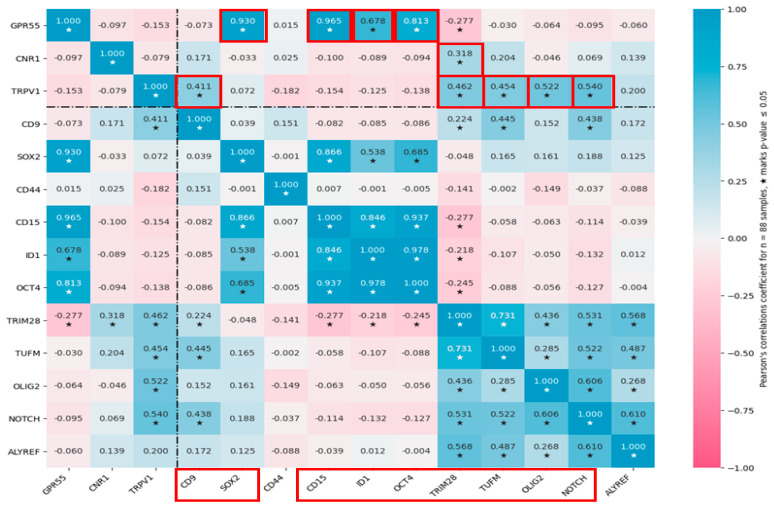
Correlations between the expressions of cannabinoid receptors and GSC markers in glioblastoma. Stemness genes are framed in red below. Correlations with cannabinoid receptors are framed in red above. *GPR55* significantly correlated with the GSC markers *SOX2*, *CD15, ID1*, and *OCT4. TRPV1* significantly correlated with *NOTCH*; *OLIG2*; and the putative markers *CD9, TRIM28*, and *TUFM* (the latter three of which are potential candidates to be classified as established stem cell markers [32,33,34]). *CNR1* only correlated (poorly) with *TRIM28*. * *p* < 0.05.

Next, we were interested in whether the expressions levels of *CNR1, GPR55*, and *TRPV1* are associated with GSC markers in the cohort of 89 GBM tissue samples (Figure 4). As expected, the mRNA levels of GSC markers *CD15* (carbohydrate antigen surface stem cell marker), *SOX2* (cell differentiation-regulating transcription factor), *ID1* (helix-loop-helix co-transcription factor), and *OCT4* (octamer-binding transcription factor 4) exhibited positive correlations.

*CNR1* poorly correlated with putative GSC marker *TRIM28* (a transcription factor) and did not correlate with any of the established GSC markers. *GPR55* strongly and significantly (*p* < 0.05) correlated with *CD15* (r = 0.97), *SOX2,* (r = 0.93), *OCT4* (r = 0.81), and *ID1* (r = 0.68). *TRPV1* correlated with *NOTCH* (r = 0.54) and *OLIG2* (r = 0.52) and poorly correlated (r < 0.50; *p* < 0.05) with the putative GSC markers *TRIM28* (r = 0.46), *TUFM* (r = 0.45), and *CD9* (r = 0.41).

Taken together, the correlation matrix shows the positive correlation among the cluster of GSC stemness biomarkers expression, whereas the three cannabinoid receptors did not exhibit any correlations among themselves, indicating their independent regulation.

### 3.5. Association of Cannabinoid Receptor Expression with MES, PN, and CL GBM Subtype Markers

**Figure 5:**
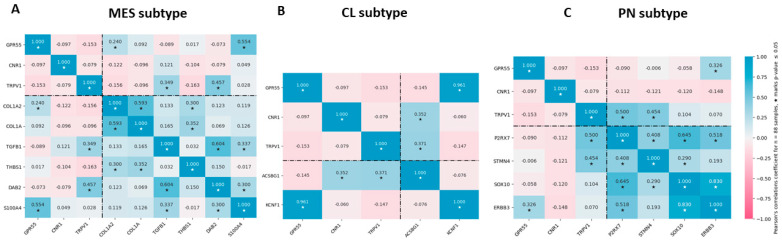
Correlation matrix of *GPR55, CNR1*, and *TRPV1* with MES, CL, and PN markers. Behnan et al. [38] suggested the following markers: (**A**) MES (*COL1A2, COL1A, TGFBI, THBS1, DAB2*, and *S100A4),* (**B**) CL (*ACSBG1* and *KCNF*), and (**C**) PN (*P2RX7, STMN4, SOX10*, and *ERBB3*). *GPR55* correlated with MES markers *COL1A2* (r = 0.24) and *S100A4* (r = 0.55), very strongly with CL marker *KCNF1* (r = 0.96), and poorly with PN marker *ERBB3* (r = 0.33). *CNR1* did not markedly correlate with any of these markers. *TRPV1* correlated with MES markers *DAB2* (r = 0.46) and *TGBF1* (r = 0.35), CL marker *ASCBG1* (r = 0.37), and most highly with PN markers *P2RX7* (r = 050) and *STMN4* (r = 045). The star under the correlation number (r) between the two genes in the boxes, means that the correlation was significant ( *p* < =0.05) n ^®^ (* *p* < 0.05).

The three major GBM subtypes, which differ in their aggressiveness, are MES, PN, and CL. We investigated whether cannabinoid receptors are associated with the biomarker expression of specific GBM subtypes [35,37] (Figure 5). *CNR1* did not markedly correlate with any of the MES or PN biomarker genes (Figure 5A,C) but did correlate with the CL *ACSBG1* gene (cyclo-CoA synthetase family member 1) (Figure 5B). *GPR55* most strongly (r = 0.96) correlated with the CL *KCF1* gene (encoding a voltage-gated potassium channel protein subunit) (Figure 5B) and less strongly with the MES gene *S100A4* (S100 calcium-binding protein A4) (Figure 5A). *GPR55* poorly correlated (r = 0.33) with the PN gene *ERBB3* (encoding an epidermal growth factor homologue) (Figure 5C). The *TRPV1* gene did not correlate with any group-specific gene patterns but positively correlated (*p* < 0.05) with the MES *TGFB1* gene (encoding *TGFβ* and *DAB2* (disabled homolog 2 protein)) (Figure 5A). *TRPV1* correlated with MES genes *DAB2* and *TGFβ* (Figure 5A), the CL gene *ACSBG1* (Figure 5B), and PN genes *P2RX7* (purinoceptor 7 protein) and *STMN4* (stathmin/oncoprotein 18 family) (Figure 5C).

### 3.6. The Cannabinoids CBG and CBD Affect the Viability of Primary GBM Cells and GSCs

**Figure 6:**
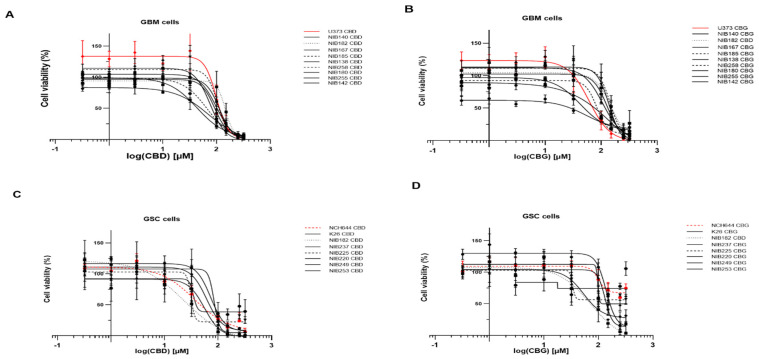
The cannabidiol (CBD) and cannabigerol (CBG) reduce viability of glioblastoma (GBM) and GBM stem cells (GSCs). CBD (**A**,**C**) and CBG (**B**,**D**) decreased the viability of ten differentiated GBM cells and eight GSCs. Dose responses of cell viability measured by MTT assay (*y*-axis) and different CBD and CBG concentrations increasing in the range of 0.32–320 µM (*x*-axis log scale) on established GBM cells (U373 line) (A, B; red line) in comparison with patient-derived primary GBM cells (A, B; black lines). Established GSC lines NCH644k (C, D; red line) are compared with patient-derived primary GSCs (C, D; black lines) after 48 h of single treatment with cannabinoids. The solvents base emulsion or DMSO (≤0.4%, *v*/*v*) were used as controls. Data are expressed as mean ± SE (*n* = 3–5 independent biological experiments, each with technical triplicates). Vehicles comprised ≤0.1% base emulsion.

Next, we investigated the effects of CBD:CBG dissolved in base emulsion, which was designed to achieve better in vivo delivery to the tumour. We treated the primary GBM and GSC cell lines with CBD and CBG at concentrations of 0.32–320 µM as described in Methods (Table 2 and Figure 6). Both cannabinoids significantly reduced the viabilities of both GBM lines (Figure 6A,B) and GSCs (Figure 6C,D). In nine patient-derived primary GBM cell lines, CBG reduced cell viability in a concentration range of IC50 100 ± 15.3 μM. Tested on the same cell lines, CBD was not significantly more cytotoxic: IC50 78.9 ± 7.8 μM (Table 2). In eight patient-derived primary GSCs, the IC50 values were 84 ± 15.3 μM for CBG and 50 ± 7.1 μM for CBD. Furthermore, our results demonstrate that CBG and CBD reduce the viability of GBM and GSC lines in the same order of magnitude (Figure S2). These quantitative results confirm previous results on the effects of CBD and CBG (dissolved in DMSO and ethanol) on GBM and GSC cell viability [31]. When comparing IC50 values of CBD and CBG in base emulsion vs. ethanol/DMSO, we found significantly higher values when using base emulsion (Figure S3).

The selected clinical data of the patients are included in Table S2.

### 3.7. CBD and CBG Cytotoxic Effects Alone and at the Molar Ratio of 3:1 on GBM and GSC Cells

CBD and CBG alone inhibited differentiated GBM cells, and CBD was slightly more effective (Table 2). CBD also had markedly stronger cytotoxic effects on GSCs than on differentiated GBM cells, which were also stronger than the effects of CBG (Figure S2). CBG was more efficient at decreasing GSC viability. These results indicate the higher sensitivity of GSCs to both cannabinoids. This effect on GSCs was particularly fortified when CBD was mixed with CBG; however, the persistent differences between GSCs and differentiated GBM cells did not reach statistical significance, mostly due to the small cohort of tested cells. Nevertheless, the effects of cannabinoids are opposite to cytotoxic drugs and irradiation treatment, to which GSCs are known to be more resistant than GBM cells.

The combined effect of CBG and CBD was tested based on our previous results [31], which showed significantly better inhibition of cell viability with CBD:CBG mixtures.

Here, we further optimized the ratio between CBD and CBG in base emulsion (Figure S3). 

Next, to possibly increase the beneficial additive effects of CBG, we increased its level in the CBD: CBG mixture from 4: 1, via 3:2 to 3:1, the most efficient molar ratio, also suggesting that higher CBG would not add to the cytotoxic effects on GBM/GSC cells

**Figure 7:**
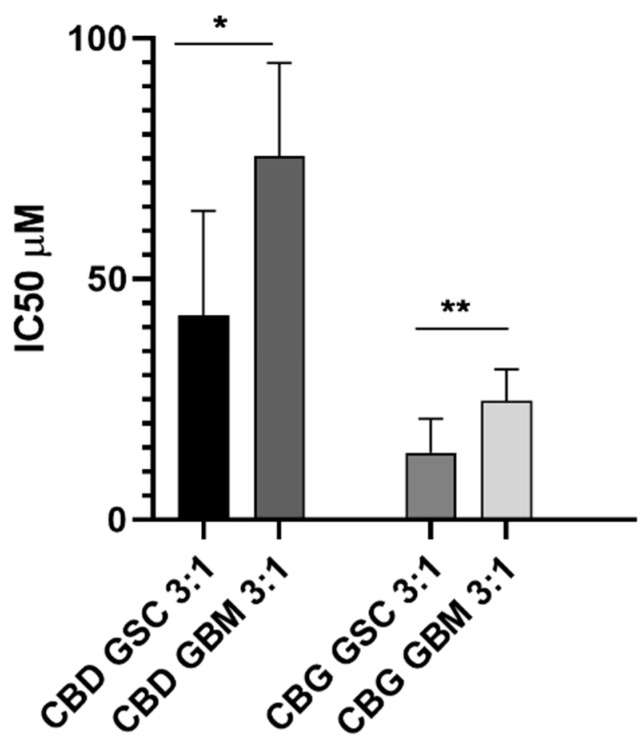
Cytotoxicity of cannabidiol (CBD) and cannabigerol (CBG) mixtures on glioblastoma (GBM) cells and GBM stem cells (GSCs). Mean IC50 values for GSCs (*n* = 8) and differentiated GBM cell lines (*n* = 10) treated with CBD and CBG (at a molar ratio of 3:1) taken from Table 2 are presented as bars of the mean cytotoxic effects, expressed in mean CBD and CBG absolute concentrations. The paired t-test was used to evaluate statistical difference (* *p* < 0.0105, ** *p* < 0.0094).

In 9 patient-derived primary GBM cell lines, the CBD:CBG mixture reduced GBM cell viability in a concentration range of IC50 76 ± 6.6:25 ± 2.1 μM. In 8 patient-derived primary GSC lines, the CBD:CBG mixture reduced GSC viability in a concentration range of IC50 42 ± 3.4:16 ± 1.1 μM (Table 2). We conclude that GSCs are significantly more sensitive to CBD:CBG mixtures (at a ratio of 3:1) than GBM cells (Figure 7).

## 4. Discussion

Three major CNRs, highly specific CB1, less selective GPR55, and non-specific ion receptor protein TRPV1, are overexpressed in low-grade glioma and GBM vs. normal brain. Here, we demonstrated their correlations with glioma grade, patient survival, and selected genetic patterns in GBMs (WHO grade IV, IDH-1 wild type). To date, no study has investigated these associations between receptor expression and the above-mentioned GBM characteristics in the same cohort of glioma and GBM tissues as well as in primary differentiated GBM cells and GSCs.

First, *CNR1* and *GPR55* mRNA expressions between different degrees of glioma malignancy, i.e., low-grade glioma vs. GBM, were not significantly different, but were higher than those in non-malignant brain (Figure 1A, B). Cell cultures revealed more significant differences in gene expression, indicating that stromal cells in the tumour microenvironment (TME) also express these receptors and may contribute to cannabinoid treatment response. Compared with GBM cells, GSCs exhibited significantly lower *CNR1* expression and higher *GPR55* and *TRPV1* expression (Figure 1). The three cannabinoid receptor genes did not correlate with each other in glioma (Figure 2) or GBM tissues (Figure 3), indicating their independent regulation. In glioma tissues, highly expressed *CNR1* and *GPR55* did not correlate with increased malignancy. Furthermore, overall survival analyses of glioma and GBM patients did not show any correlation between survival and *CNR1* expression. Conversely, *TRPV1* was significantly downregulated with increased glioma grade (Figure 2, Table 1).

With respect to 12 biomarkers [38] related to MES, CL, and PN GBM subtypes, analysis of GBMs [35] showed no correlation to receptor expression and consequently no relation to the pathophysiology of the three GBM subtypes (Figure 5). This could be explained by high GBM intra-tumour heterogeneity, which prevents any subtype gene pattern from predominating. For example, *TRPV1* correlated with the MES subtype markers *DAB2* and *TGFB1*, CL marker *ASCBG1*, and the PN markers *P2RX7* and *STMN4.* These correlations between *TRPV1* and stemness genes are discussed below.

### 4.1. Specific Cannabinoid Receptors in Glioma Progression

*CNR1* expression was much higher than often even undetectable *CNR2* expression (not shown). Our previous study demonstrated the presence of CB2 protein in tumour tissues, cells, and GSC spheroids by immunochemical and immunofluorescent analyses [34]. Similarly, Held-Feindt et al. [22] showed CB1/*CNR1* overexpression in malignant brain at the protein and gene levels, whereas CB2/*CNR2* expression was much lower and even below the detection limit in both malignant and normal brain. Kolbe et al. [24] also reported highly variable expressions of CB1 and CB2 in primary GBM cells, observing low *CNR2* gene expression but not always low CB2 protein expression. Over-expressed levels of specific CNRs are expected in all cancer cells [39] due to the well-documented high anticancer activity of the endogenous endocannabinoid system in the brain [13,40], which undoubtedly plays a role in glioma malignancy. For example, in GBMs expressing high CB1 level, CBD counteracted tumour growth also by inhibiting the endocannabinoid anandamide-degrading enzyme, thereby enhancing endocannabinoid activity.

### 4.2. The Expression and Potential Role of GPR55 in Glioma

G-protein-coupled receptor GPR55 (CB3) [40], also called orphan receptor, reportedly promotes cancer cell proliferation, both in cell cultures and in xenografted mice, and has thus become an important new biomarker and therapeutic target. We found that *GPR55* was overexpressed in GBM compared with non-malignant brain (Figure 1B) but exhibited no correlation with tumour grade or patient survival (Figure 2, Table 1). At median concentration and higher cut-offs and with disease progression, the risk of death increases, whereas *GPR55* expression was not relevant for survival. We also confirmed that *GPR55* at the median cut-off concentration was not significant for survival in GBM patients from the TCGA GLIOVIS data bank (Figure 3D).

Andradas et al. [41,42] showed that *GPR55* expression correlated with the aggressiveness of breast cancer, pancreatic carcinoma, and GBM. Moreover, by over-activating the MAPK/ERK cascade, *GPR55* promoted cancer cell proliferation, observed as higher Ki67 expression, in both cell cultures and xenografted mice. These authors also showed that the lower overall survival rate in 74 high-grade glioma patients correlated with higher *GPR55* expression. This is not consistent with our data, which demonstrate here no such correlation in 86 GBM patients. These data are difficult to interpret, supporting the findings of Kolbe et al. [24] regarding enigmatic, scattered, differential *GPR55* activity in primary GBM cells. The authors highlighted the high heterogeneity of GBMs regarding CNR signalling pathways and concluded that if cannabinoids are to be considered as additional therapeutic agents, their efficacy must be evaluated in each patient. The GPR55 antagonistic activity of CBG and recently confirmed CBG affects proliferation via cycle arrest in the G1 phase [34].

Another undefined variable is related to the fact that GPR55 forms heterodimers with CB2 and TRPs, significantly changing the kinetics of cannabinoid binding [20,43,44]. This may be in line with our data that show that *GPR55* is very strongly correlated with the genes *S100A4* and *KCNF* (Figure 5B), encoding calcium-binding protein and ion voltage protein transporter, respectively. The latter protein most likely forms heterodimers with GPR55. In this context, Kolbe et al. [24] emphasized that distinct signalling pathways are activated by GPR55 vs. other CNRs. Another relevant observation is that *GPR55* was markedly more expressed in GSCs than GBM cells. This association is supported by a strong correlation with a distinct pattern of GBM stemness genes, of which *CD15* has the strongest correlation factor (r), followed by *SOX2, OCT4,* and *ID1.* By contrast, no correlation between selective receptor *CNR1* and GSC stemness genes was observed (Figure 4).

**Scheme 1:**
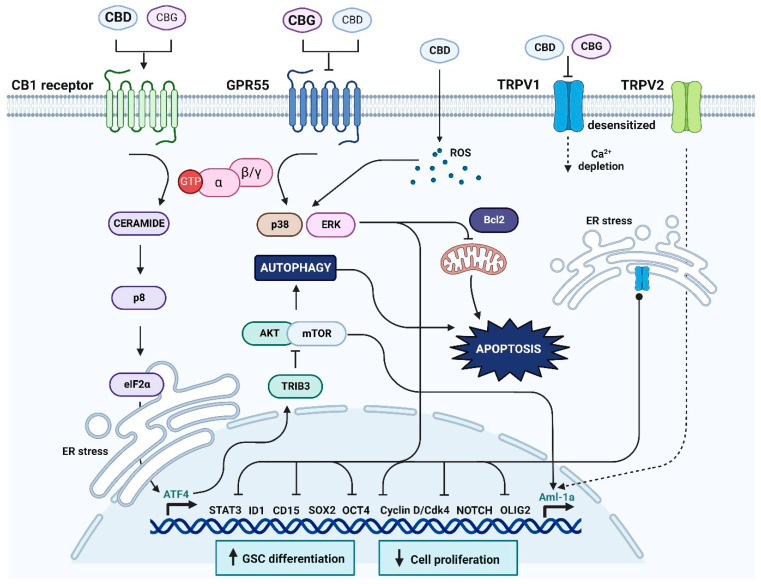
The cannabinoids cannabidiol (CBD) and cannabigerol (CBG) affect glioblastoma tumour differentiated d-glioblastoma cells and glioblastoma stem cells (GSCs).

Taken together, we speculate that both CBD and CBG may induce cell differentiation due to their relatively stronger effects on GSCs via GPR55 signalling suppression that also suppresses GSCs stemness genes. This hypothesis must be further confirmed; however, it corroborates all previous studies on cannabinoids and CNR ligands that demonstrated GSC differentiation [12,13,14]; however, these studies did not mention the potential role of GPR55 (Scheme 1).

Treatment with the mixture of CBD:CBG induces two parallel mechanisms in GSCs and differentiated GBM cells. First, due to the higher GPR55 and TRPV1 expression in GSCs, the two cannabinoids bind preferentially, although with different affinities, to antagonise GPR55, desensitize TRPV1 on GSC cells, and trigger cell differentiation processes by downregulating stemness genes. This also inhibits proliferation and induces the autophagy and apoptosis of both types of glioblastoma cells. The simplified, well-known signalling pathways related to CB1, GPR55, and TRPV1 receptors upon cannabinoid binding are linked via various common intermediate markers, as follows:

(I) The binding of CBD to allosteric sites on the CB1 receptor is stronger than that of CBG and triggers ER (endoplasmic reticulum) stress; according to Peeri and Koltai [36], this stimulates the synthesis and accumulation of ceramides, possibly via G-coupled proteins, inducing several pathways. First, upregulated protein p8 triggers post-ER stress factors, e.g., eIFKA3 (eukaryotic translation initiation factor 2-alpha kinase 3), which is increased in CBD-treated GSCs [13]. This is linked to activating transcription factor-4, inducing activating transcription factor-3 and the synthesis of triple homologue 3, a pseudo kinase, which inhibits the cytosolic Akt-kinase. The inhibition of Akt and the associated mTOR complex initiates autophagy, which may lead to apoptosis. The second pathway related to Atk/mTOR inhibition upregulates/activates several apoptosis-related B-cell lymphoma-2 family genes/proteins.

(II) CBD/CBG inactivate the highly overexpressed GPR55 signalling in GSCs, which would normally promote GBM proliferation via p38/MAPK/ERK kinase. This is followed by the activation of several transcription factors that mostly activate cell-cycle-related genes (cyclin D/Cdk4 kinase) and cell proliferation. This cannabinoid-induced pathway inhibition leads to cell cycle arrest and induces the intrinsic, mitochondria-mediated, caspase-3/7-activated apoptotic pathway. Finally CBD non-receptor transmembrane diffusion was reported to induce reactive oxygen species, and p38/MAPK activation underlies the reactive oxygen species-mediated reprogramming of GSCs [15]. Singer et al. [45] showed that stem cell key regulators, e.g., STAT3, ID1, and SOX2, are inhibited by CBD under such conditions. These stemness genes, including *OCT4* and *CD15*, highly correlate with *GPR55*, as found in the presented (Figure 4).

(III) The role of TRPV1 at plasma and ER membranes is to regulate Ca^2+^ influx/efflux. Additionally, when TRPV1 is desensitised by CBD/CBG binding, it also induces intrinsic pro-apoptotic mechanisms [46] via Ca^2+^ depletion, triggering ER stress. We found significantly higher expression of *TRPV1* in GSCs as in GBM cells (Figure 1C), and a correlation between *TRPV1* and *NOTCH* and *OLIG2* and other GSC stemness and epithelial-to-mesenchymal transition markers. Thus, we hypothesize that TRPV1 signalling is also essential to maintain GSCs, and when agonized, it is desensitized by CBD and CBG cannabinoids, triggering Ca^2+^ depletion in ER that may also lead to GSC differentiation. The involvement of TRPV2 and Akt kinase was associated with the transcription of acute myeloid leukaemia [14]. Taken together, we confirmed the notion that a well-defined combination of pure cannabinoids is advantageous over single agents [47]. Although their optimal ratio seems to depend on the nature of cancer cells [24], even heterogenous GBM cells, we were able to optimize the ratio to better target a cohort of GSCs vs. GBM cells.

### 4.3. The Expression and Potential Role of Non-Selective TRPV1 in Glioma

Different phytocannabinoid signalling occurs via TRPV1, a non-selective Ca^2+^-permeable channel transporter that is central to a plethora of cancer-associated processes [48]. In this study, in contrast to *CNR1* and *GPR55*, *TRPV1* mRNA negatively correlated with glioma grade and positively correlated with overall survival (Figure 1 and Figure 2, Table 1). This indicates its tumour suppressor-like regulation. This observation is in line with its observed suppression of other cancers, e.g., lung [25] and skin [49], and intestinal carcinoma [50] well as melanoma growth [51]. TRPV1 is a target of its activator capsaicin (trans-8-methyl-N-vanillyl-6-nonenamide) and is also responsive to endocannabinoids [30]. In contrast to capsaicin, CBD and CBG activation do not induce very large, permeant-pore-dilated channels but rather differentially activates them and even desensitizes TRPV1, depending on external Ca^2+^ concentrations [52]. The perturbed Ca^2+^ influx and efflux between the cytosol, mitochondria, and ER result in the release of apoptosis-inducing factor and cytochrome c from the mitochondria and caspase activation, followed by DNA fragmentation (Scheme 1).

Moreover, *TRPV1* is significantly more overexpressed than *GPR55* in primary GSCs (Figure 1C) vs. GBM cells. *TRPV1* was correlated with stemness-related genes, most strongly with *NOTCH* and *OLIG2*, followed by the putative GSC stemness genes *CD9* [53], *TRIM* [54], and *TUFM* [55] (Figure 4). *TRPV1* was also correlated with some characteristic genes related to the epithelial-to-mesenchymal transition, e.g., *NOTCH, NFKB1*, *TGFB1*, and *CDH1*, but not with *SNAIL* and *VIM* (Figure S1). However, this is not unusual, as the epithelial-to-mesenchymal-like transition of GBM cells proceeds via various signalling pathways, acting synergistically or energetically through an intermediate epithelial–mesenchymal state. *SOX2* and *OLIG4* expressions [8] are in meta-equilibrium between differentiated and undifferentiated stem-like states, with temporarily increased stemness genes, which may or may not even proceed to complete the full mesenchymal transdifferentiation [56]. Our data allow the speculation that *TRPV1* expression may also be associated with intermediate epithelial-to-mesenchymal and mesenchymal-to-epithelial stem like-states with overexpressed *TGFB1* and *NOTCH* genes.

Initially, Aguado et al. [57] demonstrated that cannabinoids promote GSC differentiation in a receptor-dependent manner, as TRPV1 was inhibited by the antagonist capsazacepin that reduces gliomagenesis in vivo. Later, Nabissi et al. [14] demonstrated that acute myeloid leukaemia 1 transcription factor, induced via TRPV2 and phosphoinositide-3-kinase/protein kinase B, affects GSC differentiation.

In our study, the significant upregulation of *TRPV1* in GSCs vs. GBM cells (Figure 1C) and the cytotoxicity, presumably induced by TRPV1 antagonists CBD and CBG (Figure 6, Table 2) strongly supports the potential role of TRPV1 in GSC differentiation. Moreover, the potential anti-GBM mechanism of TRPV1 involvement has already been revealed by Stock et al. [58], who showed high *TRPV1* expression in neural progenitor cells during embryonal neurogenesis. In GBM, these neural progenitor cells may be activated to migrate and interact with high-grade astrocytomas, release certain fatty-acid ethanolamide agonists to TRPV1, reduce glioma expansion, and prolong survival by stimulating tumour-supressed TRPV1. This might deplete Ca^2+^ stores and induce eukaryotic initiation factor 2 alpha and thus transcription factor-3 and -4, in turn leading to GBM/GSC death via the ER stress pathway, as discussed in [13] and shown in Scheme 1.

### 4.4. Cannabinoids CBD and CBG Inhibit Viability of Glioblastoma Stem Cells

A vast body of literature on the anti-cancer effects of non-THC in vitro and in vivo has accumulated [59]. In the continuation of previous research [31], we studied optimised CBD and CBG combinations to omit psychoactive THC, as a vast body of literature on the anti-cancer effects of non-THC cannabinoids in vitro and in vivo has accumulated [59]. Here, we demonstrate viability inhibition by emulsion-embedded CBD and CBG (Figure S3), comparing the two cohorts of nine primary GBM and eight GSCs lines (Figure 7, Table 2). We confirmed the slightly higher cytotoxicity of CBD than of CBG, but both were more cytotoxic to GSCs than to differentiated GBM cells. Optimizing the combination of the two cannabinoids (3 CBD:1 CBG) [34] resulted in a significantly (two-fold) higher cytotoxicity to GSCs than two GBM cells (Figure 7). Based on our previous work [34], we propose a mechanism in which adding CBG arrests the cell cycle in the G1/S1 and G2/M phases, facilitating apoptosis via cytostatic effects to a greater extent than CBD (Scheme 1). Similar has been recently reported in cholangiocarcinoma [46]. A decrease in the number of cholangiocarcinoma cells in the S stage of the cell cycle and a significant increase in late-stage apoptosis were observed after combined CBD:CBG vs. single cannabinoid treatment. Moreover, CBD and CBG treatments significantly inhibited cell migration, invasion, and colony formation in both cholangiocarcinoma and GBM [34]. These effects may be predominantly due to the CBG-induced inhibition of GPR55, affecting proliferation by impairing MAPK/ERK signalling. Conversely, CBD and CBG also activate CB1 signalling in differentiated GBM via ceramide accumulation, triggering ER stress, which leads to apoptosis by two pathways: autophagy and mitochondria/executive caspase activation (Scheme 1). The proposed mechanism involves the induction of GSC differentiation via the binding of both CBD and CBG to GPR55 and TRPV1. This leads to the conclusion that only targeting CB1 in GBMs to prevent recurrence would not be efficient, as GSC differentiation is involved as well.

## 5. Conclusions

Several topics are addressed here, summarised as the major conclusions:

Efficacy of CBD and CBG on glioblastoma (GBM) and GBM stem cells (GSCs):

The translational clinical value of the presented study, searching for non-THC cannabinoid adjuvant therapy in GBM patients, is the determined effective CBD CBG mixture that significantly better targets primary GSCs as differentiated GBM cells. In future, this formulation is suggested to be validated in vivo in a larger cohort of animal studies and in adjuvant to standard-of care GBM therapy.

Relative expression of CB1/CNR1, GPR55 and ionotropic TRPV1 genes in GBM and GSCsb shows similar levels of *CNR1* gene but markedly higher levels of *GPR55* and significantly higher levels of *TRPV1* genes in GSCs. Accordingly, the cytotoxicity of CBD and CBG on GSCs was significantly stronger and reportedly includes the binding and activation of CB1, the inhibition of GPR55 receptors, and the agonistic modulation of TRPV1 in GBM. The hypothetical mechanism is presented in Scheme 1, comprising all state-of-the-art information on the endocannabinoid system in glioblastoma cells and stem cells. In short, CBD and CBG activate CB1 signalling in differentiated GBM via ceramide accumulation, triggering ER stress, which leads to apoptosis by two pathways, i.e., via autophagy or mitochondria/executive caspase activation. The specific role of CBG seems to be mostly the inhibition of GPR55, affecting proliferation by impaired MAPK/ERK signalling.

The relative expression of CNR1, GPR55 and TRPV1 genes in tissues showed that the three cannabinoid receptor genes did not correlate to each other in glioma or in GBM tissues, meaning they were independently regulated. Highly expressed *CNR1* and *GPR55* genes in glioma and GBM vs. non-cancerous brain tissues did not correlate with increased malignancy or GBM subtype, respectively. In contrast, the ionotropic receptor *TRPV1* gene was significantly downregulated with glioma progression, possibly indicating its tumour suppressor role. However, overall survival analyses in the cohort of 86 GBM patients, did not show any correlation between survival and receptors’ expression. The difference in tissue vs. isolated tumour cells clearly points to the receptors/endocannabinoid system activity in the stromal cells, comprising tumour microenvironment.

Correlations of the cannabinoid receptors with molecular GBM/GSCs biomarkers.

The highly relevant finding of this study is the selective and specific correlations among different sets of the stemness and epithelial-to-mesenchymal transition-related genes. Based on these correlations and the literature data, we propose a simplified signalling scheme of preferential targeting of GSCs cells via antagonizing GPR55 and inversely agonizing TRPV1 receptors, which are on one hand both highly expressed in GSCs and on the other correlate with several major GBM stemness genes (*CD15, SOX2, OLIG4, NOTCH*, and *ID1*). The GSC differentiation processes may be at least partially induced by downregulating these stemness genes, followed by inhibiting proliferation and inducing autophagy and apoptosis. However, these mechanisms need to be confirmed by selectively regulating the mRNA of the receptors and stemness proteins and observing the effects in cannabinoid treatment on their expression, which were beyond the scope of this report. Additionally, due to the above-described ECS signalling complexity, the differential expression of cannabinoid receptors in GBM and GSC cells, and the CBD and CBG interactions with their receptor network in a single tumour/patient, it is difficult to explain in more details and to predict their effects.

Further studies should also be related first to the complex GBM microenvironment, as it has been well established that the mechanism of cannabinoid receptor-induced anti-tumour activity in experimental glioblastoma animal models is more complex than in in vitro models and involves an inhibition of not only cancer cells survival/proliferation, but also tumour invasiveness, angiogenesis and immune response [13,15], involving infiltrated stromal cells. Secondly, studies related to normal brain cells, neurons an astrocytes, comprising the major part of GBM microenvironment reported on lively cross talk with GBM, but these stromal cells are not affected by cannabinoids, as reviewed in vitro [23,60] and shown in clinical study in vivo [61].

## Figures and Tables

**Table 1:** Significance of correlations in Figure 2A.

*p*-Value							
Sample Type	0						
Survival	<0.001	0					
Death	0.012	<0.001	0				
WHO grade	<0.001	<0.001	<0.001	0			
GPR55	0.144	0.258	0.903	0.219	0		
CNR1	0.054	0.397	0.128	0.187	0.701	0	
TRPV1	0.132	**0.004**	**0.012**	**0.004**	0.712	0.164	0
	Sample Type	Survival	Death	WHO grade	GPR55	CNR1	TRPV1

The data represent significance in *p*-values among the receptors and clinical variables (*p* < 0.05 is considered significant). The correlations between the variables are presented as Pearson correlation coefficients (r), which measure the linear relationship between two sets of variables. In Figure 2A, the magnitude of the coefficient, represented by the number and diameter of the circle, indicates the association between the two variables. Bold *p* values are related to the significance of negative correlations.

**Table 2:** Mean IC50 (µM) values for glioblastoma (GBM) differentiated and GBM stem cells (GSCs) for cannabidiol (CBD) and cannabigerol (CBG) treatment alone and at the molar ratio of 3:1.

GBM Cell Lines	IC50 Values (μM)	CBG	CBD:CBG = 3:1
U373 ^1^ (GB)	99	61	68:22
Primary patients derived at NIB			
NIB140 (GB)	114	175	79:27
NIB142 (GB)	71	53	80:26
NIB138 (GB)	61	54	46:15
NIB180 (GB)	83	127	98:32
NIB185 (GB)	104	144	103:34
NIB182 (GB)	47	156	95:31
NIB167 (GB)	99	107	71:23
NIB258 (GB)	68	83	65:21
NIB255 (GB)	43	42	50:16
Means	78.9 ± 7.8	100 ± 15.3	76 ± 6.6:25 ± 2.1
GSC lines			
NCH644 ^2^ (GSC)	34	100	34:11
Primary patients derived at NIB			
K26 (GSC)	73	138	54:18
NIB216 (GSC)	24	90	30:10
NIB237 (GSC)	50	132	27:9
NIB225 (GSC)	50	34	28:9
NIB220 (GSC)	84	98	38:12
NIB249 (GSC)	52	61	46:15
NIB253 (GSC)	35	18	30:10
Means	50 ± 7.1	84 ± 15.3	42 ± 3.4:16 ± 1.1

(^1^) U373: established, commercial cell line (American Type Cell Collection, Manassas, VA, USA). (^2^) NCH644 lines: established, commercial cell lines (CLS-Cell Lines Service GmbH, Eppelheim, Germany).

## Data Availability

All the data, supporting reported results can be provided upon request from the corresponding authors.

## Supplementary Materials

The following supporting information can be downloaded at: https://www.mdpi.com/article/10.3390/cancers14235918/s1, Figure S1: Correlation matrix of GPR55, TRPV1 and CNR1 with EMT markers; Figure S2: Mean IC50 values for GSC and GB cell lines treated with CBD and CBG, dissolved in BE. Figure S3: Mean IC50 values for GSC and GB cell lines treated with CBD and CBG, dissolved in EtOH/DMSO and BE. Table S1: List of assays used for RT-qPCR analysis (Thermo Fisher Scientific, USA), Table S2. Details of the GBM patients operated at the Department Neurosurgery of the University Medical Centre Ljubljana, Slovenia, used for cell viability assays.

## Abbreviations

ACSBG1	Acyl-CoA Synthetase Bubblegum Family Member 1
AIF1	Allograft Inflammatory Factor 1
ALYREF	Aly/REF Export Factor
AML-1	Acute Myeloid Leukemia 1
ATCC	American Type Cell Collection
ATF3	Activating Transcription Factor 3
ATF4	Activating Transcription Factor 4
BCL2	B-cell Lymphoma 2
BE	Base Emulsion
bFGF	Beta Fibroblast Growth Factor
CAPSAICIN	Trans-8-methyl-N-vanillyl-6-nonenamide
CB1	Cannabinoid Receptor Type 1
CB2	Cannabinoid Receptor Type 2
CBD	Cannabidiol
CBG	Cannabigerol
CCA	Cholangiocarcinoma
CCL5	Chemokine (C-C motif) ligand 5
CCR3	C-C Motif Chemokine Receptor 3
CCR5	C-C Motif Chemokine Receptor 5
CD15	Lewis X Antigen
CD44	CD44 molecule
CD9	CD9 Antigen
CDH1	Cadherin 1
CEBPA	CCAAT Enhancer Binding Protein Alpha
CHI3L1	Chitinase-3-like protein 1; YKL-40
CL	Classical
CLS	Cell Lines Service
CNR1	Cannabinoid Receptor 1 gene
CNR2	Cannabinoid Receptor 2 gene
CNRs	Cannabinoid Receptors
COL1A1	Collagen Type I Alpha 1 Chain
COL1A2	Collagen Type I Alpha 2 Chain
CSCs	Cancer Stem Cells
CST7	Cystatin F gene
DAB2	DAB Adaptor Protein 2
DMEM	Dulbecco’s Modified Eagle Medium
DMSO	Dimethyl sulfoxide
DPYSL2	Dihydropyrimidinase Like 2
eCB	Endogenous Cannabinoid-like Ligands
ECS	Endocannabinoids System
EGF	Epidermal Growth Factor
EGFR	Epidermal growth factor receptor
eIF2α	Eukaryotic Initiation Factor 2 alpha
eIFKA3	Eukaryotic Translation Initiation Factor 2-alpha kinase 3
EMT	Epithelial-to-Mesenchymal Transition
ER	Endoplasmic Reticulum
ERBB3	Erb-B2 Receptor Tyrosine Kinase 3
EtOH	Ethanol
FBS	Foetal Bovine Serum
FREM2	FRAS1 Related Extracellular Matrix 2
FUT4	Fucosyltransferase 4
GAPDH	Glyceraldehyde 3-phosphate dehydrogenase
GBM	Glioblastoma
GBM rec	Recurrent GBM
GFAP	Glial Fibrillary Acidic Protein
GPCRs	G protein-Coupled Receptors
GPR55	G Protein-Coupled Receptor 55
GSC	GBM stem cell
HLH	helix-loop-helix
HPRT1	Hypoxanthine Phosphoribosyltransferase 1
ID1	Inhibitor of DNA Binding 1
KCNF1	Potassium Voltage-Gated Channel Modifier Subfamily F Member 1
LGG	Low Grade Glioma
MAPK/ERK	Mitogen-activated protein kinases/extracellular signal-regulated kinases
MES	Mesenchymal
mRNA	Messenger RNA
MTS	3-(4,5-dimethylthiazol-2-yl)-5-(3-carboxymethoxyphenyl)-2-(4-sulfophenyl)-2H-tetrazolium salt
MTT	3-(4,5-dimethylthiazol-2-yl)-2,5-diphenyltetrazolium-bromide
N	Non-malignant brain tissues
NA	Normal Astrocytes
NF-kB	Nuclear factor-κB
NFKB1	Nuclear Factor Kappa B Subunit 1 gene
NIB	National Institute of Biology
NOTCH1	Neurogenic locus notch homolog protein 1
NSCs	Neural Stem Cells
OCT4	Octamer-binding transcription factor 4
OLIG2	Oligodendrocyte Transcription Factor 2
P2RX7	Purinergic Receptor P2X 7
PDGFR	Platelet-derived growth factor receptor
PI3K/Akt	Phosphoinositide-3-kinase/Protein kinase B
PN	Proneural
POU5F1B	POU Class 5 Homeobox 1B; OCT4-PG1 (OCT4 pseudogene)
PROM1	prominin-1; CD133 antigen
ROS	Reactive Oxygen Species
S100A4	S100 Calcium Binding Protein A4
SNAI1	Snail Family Transcriptional Repressor 1
SOX10	SRY-Box Transcription Factor 10
SOX2	SRY-Box Transcription Factor 2
SPRY	Sprouty RTK Signaling Antagonist 1
STAT3	Signal Transducer And Activator Of Transcription 3
STMN4	Stathmin 4
TCGA	The Cancer Genome Atlas
TGFB1	Transforming growth factor beta 1 gene
TGFβ	Transforming growth factor beta
THBS1	Thrombospondin 1 gene
THC	Δ9-Tetrahydrocannabinol
TMZ	Temozolomide
TRIB3	Trible Homologue 3
TRIM28	Tripartite Motif Containing 28
TRPs	Transient Receptor Potential Channels
TRPV1	Transient Receptor Potential Cation Channel Subfamily V Member 1
TRPV2	Transient Receptor Potential Cation Channel Subfamily V Member 2
TUBB3	Tubulin Beta 3 Class III
TUFM	Tu Translation Elongation Factor, Mitochondrial
VIM	Vimentin

## References

[1] IARC: Home. https://www.iarc.who.int/.

[2] World Cancer Report—IARC. https://www.iarc.who.int/world-cancer-report-content-overview/.

[3] Philips A., Henshaw D.L., Lamburn G., O’Carroll M.J. (2018). Brain tumours: Rise in glioblastoma multiforme incidence in England 1995-2015 Suggests an Adverse Environmental or Lifestyle Factor. J. Environ. Public Health.

[4] Louis D.N., Perry A., Wesseling P., Brat D.J., Cree I.A., Figarella-Branger D., Hawkins C., Ng H.K., Pfister S.M., Reifenberger G. (2021). The 2021 WHO Classification of Tumors of the Central Nervous System: A summary. Neuro-Oncology.

[5] Weller M., van den Bent M., Preusser M., le Rhun E., Tonn J.C., Minniti G., Bendszus M., Balana C., Chinot O., Dirven L. (2021). EANO guidelines on the diagnosis and treatment of diffuse gliomas of adulthood. Nat. Rev. Clin. Oncol..

[6] Park J., Shim J.K., Yoon S.J., Kim S.H., Chang J.H., Kang S.G. (2019). Transcriptome profiling-based identification of prognostic subtypes and multi-omics signatures of glioblastoma. Sci. Rep..

[7] Sottoriva A., Spiteri I., Piccirillo S.G.M., Touloumis A., Collins V.P., Marioni J.C., Curtis C., Watts C., Tavare S. (2013). Intratumor heterogeneity in human glioblastoma reflects cancer evolutionary dynamics. Proc. Natl. Acad. Sci. USA.

[8] Gimple R.C., Yang K., Halbert M.E., Agnihotri S., Rich J.N. (2022). Brain cancer stem cells: Resilience through adaptive plasticity and hierarchical heterogeneity. Nat. Rev. Cancer.

[9] Lathia J.D., Mack S.C., Mulkearns-Hubert E.E., Valentim C.L.L., Rich J.N. (2015). Cancer stem cells in glioblastoma. Genes Dev..

[10] Biserova K., Jakovlevs A., Uljanovs R., Strumfa I. (2021). Cancer Stem Cells: Significance in Origin, Pathogenesis and Treatment of Glioblastoma. Cells.

[11] Alves T.R., Lima F.R.S., Kahn S.A., Lobo D., Dubois L.G.F., Soletti R., Borges H., Neto V.M. (2011). Glioblastoma cells: A heterogeneous and fatal tumor interacting with the parenchyma. Life Sci..

[12] Aguado T., Carracedo A., Julien B., Velasco G., Milman G., Mechoulamluis R., Alvarez L., Guzmán M., Galve-Roperh I. (2007). Cannabinoids induce glioma stem-like cell differentiation and inhibit gliomagenesis. J. Biol. Chem..

[13] Costas-Insua C., Guzmán M. (2022). Endocannabinoid signaling in glioma. Glia.

[14] Nabissi M., Morelli M.B., Amantini C., Liberati S., Santoni M., Ricci-Vitiani L., Pallini R., Santoni G. (2015). Cannabidiol stimulates AML-1a-dependent glial differentiation and inhibits glioma stem-like cells proliferation by inducing autophagy in a TRPV2-dependent manner. Int. J. Cancer.

[15] Dumitru C.A., Sandalcioglu I.E., Karsak M. (2018). Cannabinoids in glioblastoma therapy: New applications for old drugs. Front. Mol. Neurosci..

[16] Hanahan D. (2022). Hallmarks of Cancer: New Dimensions. Cancer Discov..

[17] dos Reis Rosa Franco G., Smid S., Viegas C. (2020). Phytocannabinoids: General Aspects and Pharmacological Potential in Neurodegenerative Diseases. Curr. Neuropharmacol..

[18] Deiana S. (2017). Potential Medical Uses of Cannabigerol: A Brief Overview. Handbook of Cannabis and Related Pathologies: Biology, Pharmacology, Diagnosis, and Treatment.

[19] Ligresti A., de Petrocellis L., di Marzo V. (2016). From Phytocannabinoids to Cannabinoid Receptors and Endocannabinoids: Pleiotropic Physiological and Pathological Roles Through Complex Pharmacology. Physiol. Rev..

[20] Moreno E., Cavic M., Krivokuca A., Canela E.I. (2020). The Interplay between Cancer Biology and the Endocannabinoid System-Significance for Cancer Risk, Prognosis and Response to Treatment. Cancers.

[21] O’Reilly E.M., Cosgrave J.M., Gallagher W.M., Perry A.S. (2022). Plant-derived cannabinoids as anticancer agents. Trends Cancer.

[22] Held-Feindt J., Dörner L., Sahan G., Mehdorn H.M., Mentlein R. (2006). Cannabinoid receptors in human astroglial tumors. J. Neurochem..

[23] Nahler G. (2022). Cannabidiol and Other Phytocannabinoids as Cancer Therapeutics. Pharm. Med..

[24] Kolbe M.R., Hohmann T., Hohmann U., Ghadban C., Mackie K., Zöller C., Prell J., Illert J., Strauss C., Dehghani F. (2021). THC Reduces Ki67-Immunoreactive Cells Derived from Human Primary Glioblastoma in a GPR55-Dependent Manner. Cancers.

[25] Liu Y., Lyu Y., Wang H. (2022). TRP Channels as Molecular Targets to Relieve Endocrine-Related Diseases. Front. Mol. Biosci..

[26] De Petrocellis L., Nabissi M., Santoni G., Ligresti A. (2017). Actions and Regulation of Ionotropic Cannabinoid Receptors. Adv. Pharmacol..

[27] Zhai K., Liskova A., Kubatka P., Büsselberg D. (2020). Calcium Entry through TRPV1: A Potential Target for the Regulation of Proliferation and Apoptosis in Cancerous and Healthy Cells. Int. J. Mol. Sci..

[28] Li L., Chen C., Xiang Q., Fan S., Xiao T., Chen Y., Zheng D. (2022). Transient Receptor Potential Cation Channel Subfamily V Member 1 Expression Promotes Chemoresistance in Non-Small-Cell Lung Cancer. Front. Oncol..

[29] de Almeida A.S., de Barros Bernardes L., Trevisan G. (2021). TRP channels in cancer pain. Eur. J. Pharmacol..

[30] Stock K., Kumar J., Synowitz M., Petrosino S., Imperatore R., Smith E.S.J., Wend P., Purfürst B., Nuber U.A., Gurok U. (2012). Neural precursor cells induce cell death of high-grade astrocytomas through stimulation of TRPV1. Nat. Med..

[31] Valeri A., Mazzon E. (2021). Cannabinoids and Neurogenesis: The Promised Solution for Neurodegeneration?. Molecules.

[32] Pagano C., Navarra G., Coppola L., Bifulco M., Laezza C. (2021). Molecular Mechanism of Cannabinoids in Cancer Progression. Int. J. Mol. Sci..

[33] Walsh K.B., McKinney A.E., Holmes A.E. (2021). Minor Cannabinoids: Biosynthesis, Molecular Pharmacology and Potential Therapeutic Uses. Front. Pharmacol..

[34] Lah T.T., Novak M., Pena Almidon M.A., Marinelli O., Žvar Baškovič B., Majc B., Mlinar M., Bošnjak R., Breznik B., Zomer R. (2021). Cannabigerol Is a Potential Therapeutic Agent in a Novel Combined Therapy for Glioblastoma. Cells.

[35] Novak M., Krajnc M.K., Hrastar B., Breznik B., Majc B., Mlinar M., Rotter A., Porčnik A., Mlakar J., Stare K. (2020). CCR5-mediated signaling is involved in invasion of glioblastoma cells in its microenvironment. Int. J. Mol. Sci..

[36] Peeri H., Koltai H. (2022). Cannabis Biomolecule Effects on Cancer Cells and Cancer Stem Cells: Cytotoxic, Anti-Proliferative, and Anti-Migratory Activities. Biomolecules.

[37] Ramšak Ž., Coll A., Stare T., Tzfadia O., Baebler Š., Van de Peer Y., Gruden K. (2018). Network Modeling Unravels Mechanisms of Crosstalk between Ethylene and Salicylate Signaling in Potato. Plant Physiol..

[38] Behnan J., Stangeland B., Hosainey S.A.M., Joel M., Olsen T.K., Micci F., Glover J.C., Isakson P., Brinchmann J.E. (2016). Differential propagation of stroma and cancer stem cells dictates tumorigenesis and multipotency. Oncogene.

[39] Iozzo M., Sgrignani G., Comito G., Chiarugi P., Giannoni E. (2021). Endocannabinoid System and Tumour Microenvironment: New Intertwined Connections for Anticancer Approaches. Cells.

[40] Peeri H., Shalev N., Vinayaka A.C., Nizar R., Kazimirsky G., Namdar D., Anil S.M., Belausov E., Brodie C., Koltai H. (2021). Specific Compositions of Cannabis sativa Compounds Have Cytotoxic Activity and Inhibit Motility and Colony Formation of Human Glioblastoma Cells In Vitro. Cancers.

[41] Brown A.J. (2007). Novel cannabinoid receptors. Br. J. Pharmacol..

[42] Andradas C., Caffarel M.M., Pérez-Gómez E., Salazar M., Lorente M., Velasco G., Guzmán M., Sánchez C. (2011). The orphan G protein-coupled receptor GPR55 promotes cancer cell proliferation via ERK. Oncogene.

[43] Navarro G., Varani K., Lillo A., Vincenzi F., Rivas-Santisteban R., Raïch I., Reyes-Resina I., Ferreiro-Vera C., Borea P.A., Sánchez de Medina V. (2020). Pharmacological data of cannabidiol- and cannabigerol-type phytocannabinoids acting on cannabinoid CB_1_, CB_2_ and CB_1_/CB_2_ heteromer receptors. Pharmacol. Res..

[44] Navarro G., Cordomí A., Brugarolas M., Moreno E., Aguinaga D., Pérez-Benito L., Ferre S., Cortés A., Casadó V., Mallol J. (2018). Cross-communication between Gi and Gs in a G-protein-coupled receptor heterotetramer guided by a receptor C-terminal domain. BMC Biol..

[45] Singer E., Judkins J., Salomonis N., Matlaf L., Soteropoulos P., McAllister S., Soroceanu L. (2015). Reactive oxygen species-mediated therapeutic response and resistance in glioblastoma. Cell Death Dis..

[46] Viereckl M.J., Krutsinger K., Apawu A., Gu J., Cardona B., Barratt D., Han Y. (2022). Cannabidiol and Cannabigerol Inhibit Cholangiocarcinoma Growth In Vitro via Divergent Cell Death Pathways. Biomolecules.

[47] López-Valero I., Saiz-Ladera C., Torres S., Hernández-Tiedra S., García-Taboada E., Rodríguez-Fornés F., Barba M., Dávila D., Salvador-Tormo N., Guzmán M. (2018). Targeting Glioma Initiating Cells with A combined therapy of cannabinoids and temozolomide. Biochem. Pharmacol..

[48] Cui C., Merritt R., Fu L., Pan Z. (2017). Targeting calcium signaling in cancer therapy. Acta Pharm. Sin. B.

[49] Bode A.M., Cho Y.Y., Zheng D., Zhu F., Ericson M.E., Ma W.Y., Yao K., Dong Z. (2009). Transient receptor potential type vanilloid 1 suppresses skin carcinogenesis. Cancer Res..

[50] De Jong P.R., Takahashi N., Harris A.R., Lee J., Bertin S., Jeffries J., Jung M., Duong J., Triano A.I., Lee J. (2014). Ion channel TRPV1-dependent activation of PTP1B suppresses EGFR-associated intestinal tumorigenesis. J. Clin. Investig..

[51] Yang Y., Guo W., Ma J., Xu P., Zhang W., Guo S., Liu L., Ma J., Shi Q., Jian Z. (2018). Downregulated TRPV1 Expression Contributes to Melanoma Growth via the Calcineurin-ATF3-p53 Pathway. J. Investig. Dermatol..

[52] Starkus J., Jansen C., Shimoda L.M.N., Stokes A.J., Small-Howard A.L., Turner H. (2019). Diverse TRPV1 responses to cannabinoids. Channels.

[53] Podergajs N., Motaln H., Rajčević U., Verbovšek U., Koršič M., Obad N., Espedal H., Vittori M., Herold-mende C., Miletic H. (2015). Transmembrane protein CD9 is glioblastoma biomarker, relevant for maintenance of glioblastoma stem cells. Oncotarget.

[54] Porčnik A., Novak M., Breznik B., Majc B., Hrastar B., Šamec N., Zottel A., Jovčevska I., Vittori M., Rotter A. (2021). TRIM28 Selective Nanobody Reduces Glioblastoma Stem Cell Invasion. Molecules.

[55] Jovčevska I., Zupanec N., Kočevar N., Cesselli D., Podergajs N., Stokin C.L., Myers M.P., Muyldermans S., Ghassabeh G.H., Motaln H. (2014). TRIM28 and β-actin identified via nanobody-based reverse proteomics approach as possible human glioblastoma biomarkers. PLoS ONE.

[56] Majc B., Sever T., Zarić M., Breznik B., Turk B., Lah T.T. (2020). Epithelial-to-mesenchymal transition as the driver of changing carcinoma and glioblastoma microenvironment. Biochim. Biophys. Acta-Mol. Cell Res..

[57] Aguado T., Palazuelos J., Monory K., Stella N., Cravatt B., Lutz B., Marsicano G., Kokaia Z., Guzmán M., Galve-Roperh I. (2006). The endocannabinoid system promotes astroglial differentiation by acting on neural progenitor cells. J. Neurosci..

[58] Stock K., Garthe A., de Almeida Sassi F., Glass R., Wolf S.A., Kettenmann H. (2014). The capsaicin receptor TRPV1 as a novel modulator of neural precursor cell proliferation. Stem Cells.

[59] Afrin F., Chi M., Eamens A.L., Duchatel R.J., Douglas A.M., Schneider J., Gedye C., Woldu A.S., Dun M.D. (2020). Can Hemp Help? Low-THC Cannabis and Non-THC Cannabinoids for the Treatment of Cancer. Cancers.

[60] McAllister S.D., Chan C., Taft R.J., Luu T., Abood M.E., Moore D.H., Aldape K., Yount G. (2005). Cannabinoids selectively inhibit proliferation and induce death of cultured human glioblastoma multiforme cells. J. Neuro-Oncol..

[61] Guzmán M., Duarte M.J., Blázquez C., Ravina J., Rosa M.C., Galve-Roperh I., Sánchez C., Velasco G., González-Feria L. (2006). A pilot clinical study of Δ9-tetrahydrocannabinol in patients with recurrent glioblastoma multiforme. Br. J. Cancer.

