# Supplementary material for: The Cytotoxic Effects of Cannabidiol and Cannabigerol on Glioblastoma Stem Cells May Mostly Involve GPR55 and TRPV1 Signalling
Source: Cancers (Basel). 2022 Nov 30;14(23):5918. doi: 10.3390/cancers14235918 (PMC9738061; doi:10.3390/cancers14235918)
Supplement: Supplementary file 1 [file cancers-14-05918-s001.zip › cancers-2021815-supplementary.pdf]

cancers

MDPI

Supplementary Materials: The Cytotoxic Effects of Cannabidiol and Cannabigerol on Glioblastoma Stem Cells May Mostly Involve GPR55 and TRPV1 Signalling

Tamara T. Lah, Bernarda Majc, Metka Novak, Ajda Sušnik, Barbara Breznik, Andrej Porčnik, Roman Bošnjak, Aleksander Sadikov, Marta Malavolta, Selma Halilčević, Jernej Mlakar and Roby Zomer

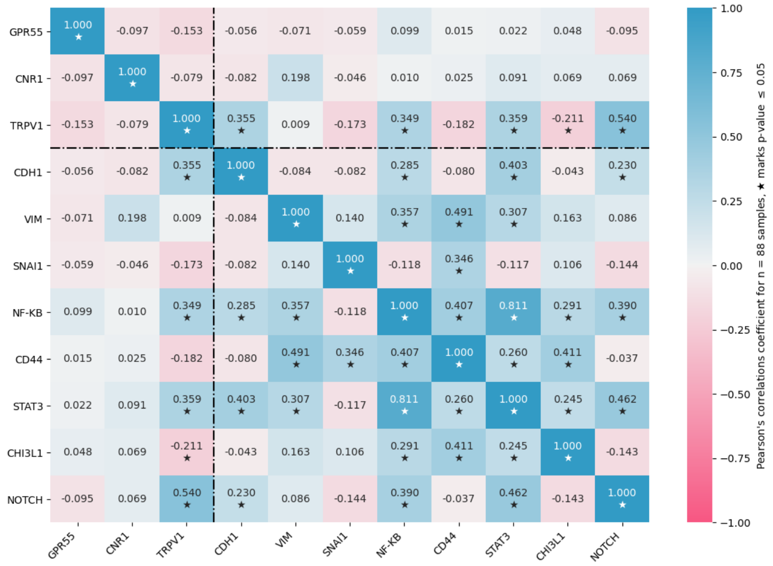

Figure S1. Correlation matrix of *GPR55*, *TRPV1*, and *CNR1* with epithelial-to-mesenchymal transition markers. These markers are *CDH1*, *vim*, *SNAI1*, *NF-Kβ*, *CD44*, *STAT3*, *CHI3L1*, and *NOTCH*. *CNR1* and *GPR55* did not significantly correlate with any of these markers. *TRPV1* correlated with *CDH1* (r=0.355), *NF-Kβ* (r=0.349), *STAT3* (r=0.359), *CHI3L1* (r=−0.211), and most strongly with *NOTCH* (r=0.540).

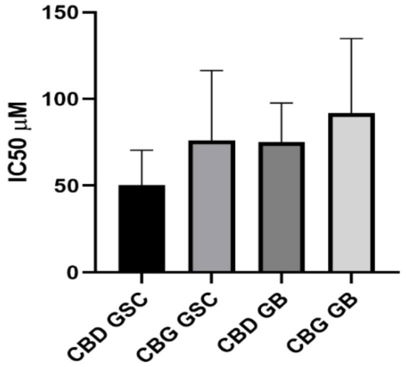

Figure S2. Mean IC50 values for glioblastoma stem cell (GSC) and glioblastoma (GB) cell lines treated with cannabidiol (CBD) and cannabigerol (CBG), dissolved in base emulsion. Bars represent the mean±SEM of the cytotoxicity of CBD on GSCs (n=8), CBG on GSCs (n=8), CBD on GB cells (n=10), and CBG on GB cells (n=10). Statistical analyses were performed with GraphPad Prism software.

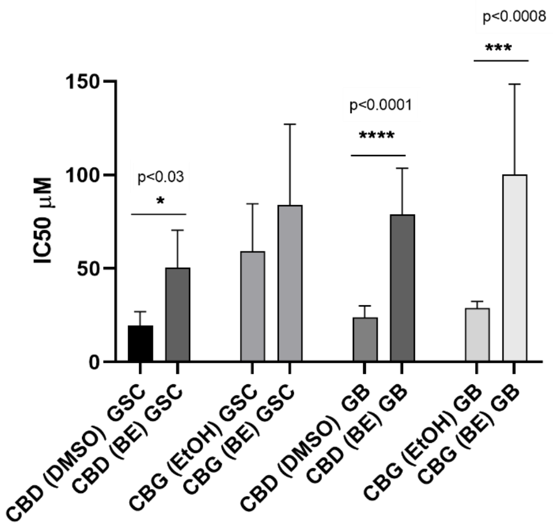

Figure S3. Mean IC50 values for glioblastoma stem cell (GSC) and glioblastoma (GB) cell lines treated with cannabidiol (CBD) and cannabigerol (CBG), dissolved in ethanol/DMSO and base emulsion. Bars represent the mean± SEM of the cytotoxicity of CBD and CBG on nine GSCs and ten GB cells. CBD/CBG in base emulsion had significantly higher IC50 values than in ethanol/DMSO. Statistical analyses were performed with GraphPad Prism software.

Table S1. List of assays used for RT-qPCR analysis (Thermo Fisher Scientific, USA).

Gene name	Protein name	Assay ID
ACSBG1	Acyl-CoA Synthetase Bubblegum Family Member 1	Hs00209500_m1
ALYREF	Aly/REF Export Factor	Hs00362329_m1
CCL5	Chemokine (C-C motif) ligand 5	Hs00982282_m1
CCR3	C-C Motif Chemokine Receptor 3	Hs00266213_s1
CCR5	C-C Motif Chemokine Receptor 5	Hs00152917_m1
CD44	CD44 molecule	Hs00174139_m1
CD9	CD9 antigen	Hs00233521_m1
CDH1	Cadherin 1	Hs01023895_m1
CEBPA	CCAAT Enhancer Binding Protein Alpha	Hs01072228_m1
CHI3L1	Chitinase-3-like protein 1; YKL-40	Hs01072228_m1
CNR1	Cannabinoid Receptor 1	Hs00275634_m1
CNR2	Cannabinoid Receptor 2	Hs00275635_m1
COL1A1	Collagen Type I Alpha 1 Chain	Hs00164004_m1
COL1A2	Collagen Type I Alpha 2 Chain	Hs01028956_m1
CST7	Cystatin F	Hs00175361_m1
DAB2	DAB Adaptor Protein 2	Hs01120074_m1
DPYSL2	Dihydropyrimidinase Like 2	Hs00265851_m1
ERBB3	Erb-B2 Receptor Tyrosine Kinase 3	Hs00176538_m1
FREM2	FRAS1 Related Extracellular Matrix 2	Hs0087621_m1
FUT4	Fucosyltransferase 4	Hs01106466_s1
GFAP	Glial Fibrillary Acidic Protein	Hs00909233_m1
GPR55	G Protein-Coupled Receptor 55	Hs00271662_s1
ID1	Inhibitor Of DNA Binding 1	Hs03676575_s1
KCNF1	Potassium Voltage-Gated Channel Modifier Subfamily F Member 1	Hs00266908_s1
NFKB1	Nuclear Factor Kappa B Subunit 1	Hs00765730_m1

NOTCH1	Neurogenic locus notch homolog protein 1	Hs01062014_m1
OLIG2	Oligodendrocyte Transcription Factor 2	Hs00377820_m1
P2RX7	Purinergic Receptor P2X 7	Hs00175721_m1
POU5F1B	POU Class 5 Homeobox 1B; OCT4-PG1 (OCT4 pseudo-gene)	Hs01596605_s1
PROM1	Prominin-1; CD133 antigen	Hs01009259_m1
S100A4	S100 Calcium Binding Protein A4	Hs00243202_m1
SNAI1	Snail Family Transcriptional Repressor 1	Hs00195591_m1
SOX2	SRY-Box Transcription Factor 2	Hs01053049_m1
SOX10	SRY-Box Transcription Factor 10	Hs00366918_m1
SPRY	Sprouty RTK Signaling Antagonist 1	Hs00398096_m1
STAT3	Signal Transducer and Activator Of Transcription 3	Hs01047578_m1
STMN4	Stathmin 4	Hs00229288_m1
TGFB1	Transforming growth factor beta 1	Hs00998133_m1
THBS1	Thrombospondin 1	Hs00962908_m1
TRIM28	Tripartite Motif Containing 28	Hs00232212_m1
TRPV1	Transient Receptor Potential Cation Channel Subfamily V Member 1	Hs00218912_m1
TUBB3	Tubulin Beta 3 Class III	Hs00801390_s1
TUFM	Tu Translation Elongation Factor, Mitochondrial	Hs00607042_m1
VIM	Vimentin	Hs00958111_m1

cancers

Table S2. Details of the GBM patients operated at the Department Neurosurgery of the University Medical Centre Ljubljana, Slovenia, used for cell viability assays.

Patient	Gender	Age (Years)	KPV	MGMT promoter methylation	Mutations				Chromosomal abnormalities
					IDH1	TP53	EGFR mutation/ amplification	Other	
**K26**	F	75	60	N.A.	wt	mut	N.A.	N.A.	N.A.
**NIB138**	F	51	90	N.A	wt	wt	-	-	-
**NIB140**	M	54	80	no	wt	wt	del eks 2-7 (EGFRvIII)	-	-
**NIB142**	F	56	80	N.A.	wt	mut	-	-	-
**NIB167**	M	84	30	no	wt	mut	-	-	-
**NIB180**	M	56	90	no	N.A.	N.A	ampl	mut pTERT C228T, del CDKN2A and CDKN2B, ampl PDGFRA, mut PDGFRA I317S, mut. AVCR1 S440G, mut. SETD2 K1706E	ampl. 7, del 10q
**NIB182**	M	74	80	yes	N.A.	N.A	-	mut pTERT C228T, PIK3R1 R631*	ampl 7, del 10q
**NIB185**	M	40	90	no	N.A.	mut R213*	-	mut pTERT C228T; C242F	ampl 7, del 10q
**NIB220**	M	56	N.A	no	wt	wt	-	mut pTERT (C228T)<br>mut PIK3CA p.(Gly118Asp) (G118D)<br>ampl EGFR, MET and PIK3CA; del CDKN2A in CDKN2B	ampl 7, del 10q, part del 1p and 19q.
**NIB237**	M	65	N.A	no	wt	mut p.(Arg267Trp) (R267W) and p.(Arg342Ter) (R342*)	-	mut pTERT (C228T).<br>mut PTEN p.(Gly36Val)(G36V).<br>FGFR1 p.(Arg455Cys) (R455C)	ampl 7, part del 1p and del 10q in 19q.
**NIB249**	M	47	N.A	no	wt	mut p.(Pro190Leu) (P190L)	mut p.(Arg108Lys)(R108K); ampl EGFR	mut pTERT (C228T) ampl MET and PIK3CA; del CDKN2A and CDKN2B<br>mut PTEN p.(Ile122Phe)(I122F).	del 10q
**NIB253**	F	58	N.A	yes	wt	del	mut p.(Arg108Lys) (R108K) and p.(Gly598Val) (G598V); ampl EGFR	mut pTERT (C228T).<br>ampl MET, MDM2, MDM4, PIK3CA, PIK3R1 and SETD2.<br>del CDKN2A, CDKN2B, FGFR1, and CIC;<br>mut PTEN p.(Glu242Gly) (E242G).	ampl 7 and del 10q in 19q.
**NIB255**	N.A	N.A	N.A	N.A	N.A	N.A	N.A	N.A	N.A
**NIB258**	M	66	N.A	no	wt	ampl	-	mut pTERT c.-124C>T (C228T).<br>mut PTEN p.(Pro248ThrfsTer5) (P248Tfs*5).<br>ampl PDGFRA, MDM2, PIK3CA, PIK3R1, ACVR1 and CIC,<br>del CDKN2A and CDKN2B.	ampl 7 and LOH 10q.

[1] Abbreviations: mut, mutated; del, deletion; part del, partial deletion; ampl, amplification; (-), No mutations identified, negative; wt, wild-type; LOH, loss of heterozygosity; N.A., Not performed.
